# Measurements of Elastic Moduli of Silicone Gel Substrates with a Microfluidic Device

**DOI:** 10.1371/journal.pone.0025534

**Published:** 2011-09-30

**Authors:** Edgar Gutierrez, Alex Groisman

**Affiliations:** Department of Physics, University of California San Diego, La Jolla, California, United States of America; German Cancer Research Center, Germany

## Abstract

Thin layers of gels with mechanical properties mimicking animal tissues are widely used to study the rigidity sensing of adherent animal cells and to measure forces applied by cells to their substrate with traction force microscopy. The gels are usually based on polyacrylamide and their elastic modulus is measured with an atomic force microscope (AFM). Here we present a simple microfluidic device that generates high shear stresses in a laminar flow above a gel-coated substrate and apply the device to gels with elastic moduli in a range from 0.4 to 300 kPa that are all prepared by mixing two components of a transparent commercial silicone Sylgard 184. The elastic modulus is measured by tracking beads on the gel surface under a wide-field fluorescence microscope without any other specialized equipment. The measurements have small and simple to estimate errors and their results are confirmed by conventional tensile tests. A master curve is obtained relating the mixing ratios of the two components of Sylgard 184 with the resulting elastic moduli of the gels. The rigidity of the silicone gels is less susceptible to effects from drying, swelling, and aging than polyacrylamide gels and can be easily coated with fluorescent tracer particles and with molecules promoting cellular adhesion. This work can lead to broader use of silicone gels in the cell biology laboratory and to improved repeatability and accuracy of cell traction force microscopy and rigidity sensing experiments.

## Introduction

Animal tissues have a broad range of elastic moduli, *E*, from <1 kPa in brain to ∼10 GPa in bone. When animal cells are grown on a substrate, they sense its rigidity, especially in the physiological range of soft tissues, with *E* = 0.1–100 kPa [1]. Variations of substrate rigidity have been shown to be important in cell migration [2], [3], development [4], [5], [6], and tumorigenesis [7], [8]. Moreover, when cells are grown on soft substrates, the cellular traction forces produce substrate deformations that are substantially large to be measured with tracer particles under a microscope. The technique of traction force microscopy (TFM) measures substrate deformations caused by cells and uses patterns of the deformations for reconstruction of maps of cell traction forces [9]. Cell traction forces are directly related to the cytoskeleton tensions and their measurements help better understand the mechanisms involved in spreading, migration, and polarization of adherent cells [1]. The most commonly used soft substrates for TFM and cell rigidity sensing studies are polyacrylamide (PAA) gels [10]. Silicone gels, such as gels based on polydimethylsyloxane (PDMS), are less widely used in biological laboratories, in spite of a long history of applications [11], [12], [13], [14], [15] and several advantages over PAA gels. Silicone gels can be stored dry, do not significantly swell in aqueous solutions, and chemical bonds in them are not susceptible to hydrolysis. In addition, silicone gels have higher refractive indices than PAA gels [16], and their surface can be patterned with a micro-relief for better tracking of the substrate deformations [13], [15].

For both TFM and cell rigidity sensing studies, it is critical to know the exact value of the elastic modulus (Young's modulus) of the gel, *E*. The elastic modulus of bulk gels can be evaluated with a variety of techniques and systems [17], from the application of tensile stresses to gel slabs using clamps and weights [10], [18], to specialized extension or compression machines [15], [19], to measurements of gel deformations under shear in rheometers [20], and to measurements of indentations produced by heavy beads [21] or use of specialized microindenters [22]. On the other hand, gel layers on cover glasses that are used in experiments on cells are commonly made to be compatible with high-resolution, short working distance microscope objective lenses, limiting the gel thickness to tens of microns. To elicit a linear response, deformations of such thin gel layers must be small. Measurements of elastic moduli of thin gel layers have been performed using micropipette aspiration [23], but the most widely used instrument for such measurements has been the atomic force microscope (AFM) [19], [24], [25]. Nevertheless, because of their high cost and considerable maintenance requirements, AFMs are poorly suited for routine tests of gel substrates prepared for experiments in a cell biology laboratory. Moreover, for optimal results, a specialized AFM probe with a microsphere glued to the tip needs to be used instead of a regular conically shaped probe [19], [26], and even then the elastic modulus of the gel is calculated from results of AFM measurements (force on the AFM probe vs. depth of indentation) using a complicated non-linear equation derived from an advanced mathematical model of gel deformation [19]. In addition, the application of AFM becomes increasingly difficult as the gel elastic modulus is reduced to ≤1 kPa, because the point of the first contact between the tip and the gel is hard to identify and because some effects not directly related to the gel elastic modulus (such as gel “stickiness” due to the attractive forces between the tip and the gel) become increasingly important [27].

The dependence of the elastic modulus of PAA gels on the molecular weight and the concentration of PAA and cross-linking agents has been tabulated by multiple groups [10], [17], [28], [29], [30]. Nevertheless, one can generally expect some variations of the gel elastic moduli due to variability of the cross-linking reaction conditions, hydrolysis, and batch-to-batch variability of the reagents. Therefore, the reliance on the published data cannot completely substitute for direct measurements of specific samples of PAA gels. The literature data on the elastic moduli of silicone gels is generally scarce, and no coherent recipe for continuous variation of the value of *E* over the physiological range of soft tissues is currently available, which is one of the likely reasons of limited use of silicone gel substrates by the cell biology community.

Here we introduce and characterize a simple technique and microfluidic setup for accurate measurements of elastic moduli of thin layers of silicone gels on cover glasses. Known hydrodynamic shear stress is applied to the surface of the gel using a microfluidic device and the resulting shear strain of the gel is measured by tracking fluorescent beads attached to the gel surface. We apply the technique to evaluate the elastic modulus of silicone gels prepared by mixing different proportions of the base (B) and cross-linker (C) components of a widely used, optically clear silicone Sylgard 184 by Dow Corning. By mixing the components B and C at ratios from 24 to 78, we found the value of *E* monotonically decreasing with the mixing ratio, B/C, from 300 to 0.4 kPa, thus covering nearly the entire physiological range. To validate the proposed technique, we independently measured the extension of slabs made of three gel samples under known tensile stresses and obtained good agreement with the data from measurements with the microfluidic device.

## Methods

### Fabrication of the microfluidic devices and gel layers

The proposed microfluidic device consists of an ∼5 mm thick polydimethylsiloxane chip (PDMS; Sylgard 184 by Dow Corning mixed at B/C = 10, with an elastic modulus of 2–3 MPa) that is sealed against a #1.5 cover glass with a 24–80 µm thick layer of silicone gel on it. The master mold to cast the chip is fabricated with a common rapid prototyping protocol, which is described in detail elsewhere [31]. Briefly, a 5 inch silicon wafer is spin-coated with a 165 µm layer of a UV-curable epoxy (SU8-2050), exposed to UV-light through a specially designed photomask, spin-coated with an additional layer of the epoxy (SU8-2100) to a total thickness of 650 µm, and exposed through another photomask, which is properly aligned with respect to the pattern generated by the first photomask. An ∼5 mm thick PDMS cast is made and cut into individual chips. Inlet and outlet holes are punched in the chips using sharpened hypodermic tubing with an internal diameter of 1/8 inch.

To prepare silicone gel substrates, 34×50 mm #1.5 microscope cover glasses are spin-coated with a gel pre-polymer (B and C components of Sylgard 184 mixed at various proportions), using a home-built spin-coater, at speeds from 1250 to 4000 rpm for gel thicknesses between 80 and 24 µm. Before the spin-coating, 40 nm carboxylated polystyrene far-red fluorescent beads (by Invitrogen, with excitation/emission maxima of 690/720 nm) are deposited on the glass surface. Gel pre-polymer is cured by baking it at 100°C for 2 hr. The gel is then treated with 3-aminopropyl trimethoxysilane for 5 min and incubated for 10 min at room temperature under a suspension of the 40 nm beads in a 100 µg/ml solution of 1-Ethyl-3-(3-dimethylaminopropyl) carbodiimide (EDC) in water to covalently link beads to the gel surface. To make a sealed microfluidic device with a silicone gel substrate, a PDMS chip is bonded to the surface of gel on a cover glass by treating the chip with oxygen plasma for 12 sec, placing the microchannel side of the chip onto the gel, and placing the cover glass with the chip on it into an 85°C oven for 15 min.

### Experimental setup and technique

**Figure 1:**
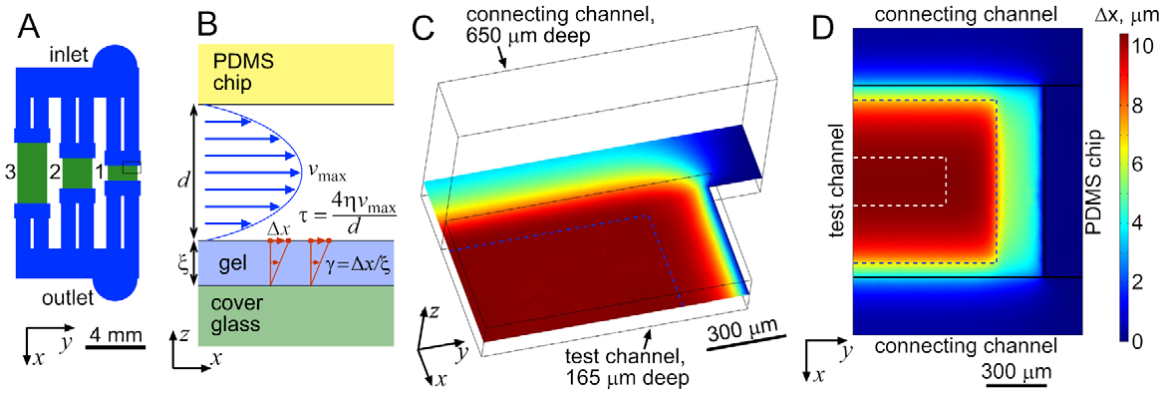
Microfluidic device, flow in it, and deformation of gel substrate under a test channel. (a) Schematic of channels in the microfluidic device. 650 µm deep connecting channels and 165 µm deep test channels are shown in blue and green, respectively. Test channels 1–3 are labeled by numbers to the left of them. (b) Schematic drawing of the *xz*-cross-section of a test channel of the device (not to scale, with fluorescent beads indicated by red dots), illustrating the proposed technique of measurements of elastic moduli of thin gel layers on cover glasses. (c) Flow velocity in the mid-plane of test channel 1 (82.5 µm from the bottom) from a numerical simulation in Comsol. The simulation domain is highlighted by a black dashed rectangle in panel A and includes the upper right quarter of test channel 1 and a fragment of the upstream connecting channel. Flow velocity is color-coded, with blue corresponding to the lowest and red corresponding to the highest values. The flow velocity is within 1% of its maximum value in the internal region to the bottom left from two blue dashed lines, which are drawn at 75 µm from the test channel entrance and at 250 µm from the right wall. Because of the symmetric layout of the channels and linear character of the flow, the flow velocity distribution in the remaining 3/4 of test channel 1 can be reconstructed by mirror-reflections about the *x*-asis at the left boundary and about the *y*-axis at the bottom boundary. (d) Color-coded map of the displacement, Δ*x*, of the top of a 70 µm thick gel layer with *E* = 2 kPa in the right half of test channel 1 and in neighboring regions under a flow with a substrate shear stress 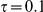 kPa from a numerical simulation in Comsol with simplified boundary conditions. Horizontal black lines are boundaries between the 165 µm deep test channel and two 650 µm deep connecting channels. 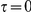 is taken in both connecting channels. Vertical black line is the boundary between the test channel and a region with gel bonded to the PDMS chip, where the condition is 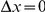. In the test channel, it is taken 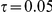 kPa outside and 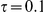 kPa inside the internal region demarcated by blue dashed lines, which is the same as the internal region marked by blue lines in panel C. White dashed lines demarcate an internal region (360 µm away from the test channel entrance and exit and 500 µm away from the side walls), in which 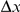 is within 1% of its maximal value (10.42 µm). The distribution of 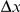 in the left half of test channel 1 can be obtained by mirror reflection about the boundary on the left.

The setup consists of the microfluidic device (Fig. 1A), a basic fluorescence microscope (Nikon Diaphot with a manual stage), and a combination of a vertical rail with a sliding stage and a regulated source of pressurized air to generate differential pressures, 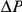, between the inlet and outlet of the device. The working fluid supplied to the device inlet and drawn out from the outlet is held in two modified 140cc plastic syringes that are connected to the device through PVC tubing with an internal diameter of 5/32 inch. (The diameter of the tubing is relatively large to minimize its flow resistance.) The value of 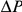 is set with an accuracy of up to 1 Pa by controlling the difference between the levels of the working fluid in the inlet and outlet syringes, 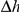, as 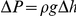, where *ρ* is the density of the fluid and *g* = 9.8 m/s^2^ is the gravitational acceleration [32]. To reach 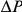
>5 kPa, the inlet syringe is connected to a source of compressed air with a pressure up to 30 kPa, which is adjusted by a sensitive regulator and measured by an electronic gauge with ∼0.25% precision.

The microfluidic device is designed to convert moderate values of 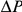 (<30 kPa) into high substrate shear stresses, *τ*, generating gel deformations sufficiently large to be reliably measured under the microscope by tracking the 40 nm fluorescent beads on the gel surface. The measured displacement of the beads, 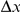, and gel thickness, 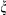, are used to calculate the shear strain in the gel, 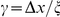 (Fig. 1B); the value of 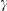 is used to calculate the shear modulus of the gel, 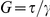, and its elastic modulus, 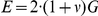, where *ν* is the Poisson ratio of the gel. Because *ν* is nearly equal to 0.5 for both PAA [33] and silicone gels [15], [34], the final equation for the elastic modulus is 
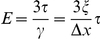
.

Importantly, the above equation for *E* follows from the first principles of continuum mechanics, is applicable to all gels, and expresses *E* in terms of the geometrical parameters, 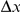 and 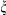, which are both readily evaluated under the microscope, and the shear stress, *τ* , which is evaluated by the analysis of flow in the microfluidic device. Furthermore, as long as flow in the device is laminar and the microchannel dimensions (specifically, their cross-sections) are unchanged, the value of *τ* in any given area of the device is proportional to 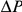 and the coefficient of proportionality, 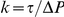, is independent of the viscosity of the working fluid flowing through the device. Therefore, once the value of *k* in a given area is established, the value of *τ* is readily calculated from the value of 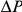, which is set and measured with a high accuracy, as described above.

The microfluidic device (Fig. 1A) has channels of two different depths, *d* = 165 µm and *d*_2_ = 650 µm. Three 165 µm deep regions of the device constitute its three test channels, where 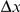 is measured as a function of *τ*. All three test channels have the same width, *w* = 2 mm. The lengths, *L*, of the test channels 1, 2, and 3 are 1, 2, and 4 mm, respectively. The 650 µm deep channels connect the test channels with the device inlet and outlet. The flow resistance per unit length, which is defined as the ratio between the pressure gradient and the volumetric flow rate, for two parallel 0.65 mm deep 1 mm wide connecting channels (Fig. 1A) is ∼39 times smaller than for a single 0.165×2 mm test channel. Therefore, the connecting channels contribute relatively little to the flow resistance between the inlet and the outlet, rendering the differential pressure across the test channels, 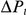, close to 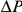, thus maximizing *τ* at given 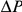.

Two major factors contributing to large values of *τ* at moderate 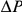 are a large ratio between the width and depth, 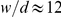, and relatively large ratios between the depth and lengths of the test channels (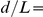
0.17, 0.085, and 0.042, respectively, for the test channels 1–3). Indeed, for a developed laminar flow in channel with a large 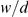, the substrate shear stress away from the channel side walls, *τ*, depends on the pressure gradient along the channel, 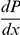, as 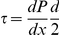, leading to an estimate of 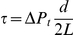 with 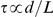 at given 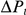.

To measure the value of *τ* in a test channel at given 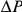, the working liquid of the microfluidic device, a 80 ∶ 20 mixture (by weight) of glycerol and water with a viscosity *η* = 0.061 Pa·s at the room temperature (20°C), is seeded with 4.6 µm green fluorescent beads. The beads are photographed under fluorescence illumination at the mid-plane of the test channels (83 µm from the substrate), where the flow velocity is maximal, and the bead streaklines are analyzed to obtain the value of the flow velocity. Numerical simulations using COMSOL (Fig. 1C) indicate that even for test channel 1, which is the shortest and has the least uniform mid-plane flow velocity, the mid-plane flow velocity varies by <1% in an internal region, which is a 850×1500 µm rectangle (*x*×*y* dimensions) with its boundaries 75 µm away from the entrance and exit of the channel and 250 µm away from the side walls (blue dashed line in Fig. 1C). Therefore, the analysis of streaklines in this internal region is expected to provide the maximal flow velocity in the channel, 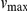, with <1% error. Moreover, the plots of the flow velocity, *v*, at heights *z* = 16.5 and 8.25 µm above the substrate in the same channel showed <1% variations of either of the two velocity fields in the same 850×1500 µm internal region (not shown), indicating nearly uniform distributions of the shear rate 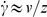 and 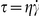. In addition, the flow velocity profile along the *z*-axis was well fitted by a parabola 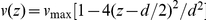. The regions of practically uniform *τ* were even larger in the 2 mm and 4 mm long test channels (not shown). Therefore, the simulations indicate that the value of *τ* can be reliably calculated from the streakline analysis as 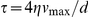 (Fig. 1B).

The high viscosity of the working fluid, *η* = 0.061 Pa·s, is instrumental for low Reynolds number, Re, in the flow. Indeed, with 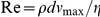 (where 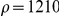 kg/m^3^ is the density of the glycerol-water mixture) and 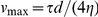, we have 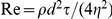, and at given 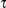, Re is proportional to 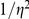. Therefore, even at the highest value of 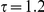 kPa we tested, Re was only ∼3, and laminar flows with 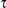 proportional to 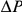 were expected in the test channels at all experimental conditions (assuming that the channel dimensions remain unchanged). Numerical simulations of the flow with the full non-linear Navier-Stokes equation, 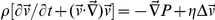, also showed practically no changes in the flow profile (flow velocity normalized to 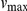) in the test channels in the entire range of 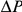.

We calculated the deformation of a gel layer with *E* = 2 kPa and 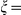
70 µm (both taken as representative values) under a shear flow in the 1 mm long test channel using a COMSOL simulation with simplified boundary conditions: uniform 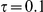 kPa in the 850×1500 µm internal region (where *τ* is nearly uniform, according to the numerical simulations of the flow), *τ* = 0.05 kPa everywhere else in the test channel, *τ* = 0 in the 650 mm deep channels, and 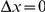 in regions under the PDMS chip (Fig. 1D). The displacement of the upper surface of the simulated gel, Δ*x*, had a maximal value of 10.42 µm, ∼0.8% less than the value of 10.5 µm given by the equation 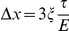. The value of Δ*x* varied by <1% in an internal region that was a 280×1000 µm rectangle (*x*×*y* dimensions) with its boundaries 360 µm away from the entrance and exit of the channel and 500 µm away from the side walls (boundaries demarcated by white dashed lines in Fig. 1D). Therefore, we expected <2% random error (and ∼0.8% positive systematic error) in the calculation of the gel elastic modulus as 
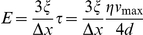
, resulting from the non-uniformity of Δ*x* and of the flow velocity in the mid-plane, when Δ*x* and 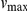 are both measured in the 280×1000 µm internal region of the 1 mm long test channel. For the 2 mm and 4 mm long test channels, the corresponding errors were estimated as <1% with negligible systematic errors (not shown).

The gel preparation technique confined the tracer particles (40 nm fluorescent beads) to two planes corresponding to the top and bottom of the gel, facilitating the measurements of *ξ*, Δ*x*, and *γ*. To measure *ξ*, the fluorescence microscope was first focused on beads on the bottom of the gel (glass surface) and then on those on the top of the gel (gel surface) and the difference in the readings of the nosepiece (*z*-axis) knob was recorded. Because the measurements were performed with a water immersion (WI) objective (60×/1.2), to calculate *ξ*, the difference was multiplied by the ratio between the refractive indices of the silicone gel and water, 1.41/1.33 = 1.06. The error in the measurements of *ξ* was estimated at ∼1 µm, which was <2% for a typical value of *ξ*≈70 µm. Because of the confinement of the tracer particles to the top of the gel, the conversion of Δ*x* into *γ*, 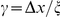, could be performed without the introduction of any new error (in addition to errors in Δ*x* and *ξ*).

The beads on the gel surface were imaged under wide-field (epi-fluorescence) illumination with minimal background that facilitated their tracking. The imaging system consisted of a 60×/1.2 WI objective, a 1× video relay lens, and a Sony XCD-900 IEEE camera with 4.65 µm pixels. With the 60× net magnification of the system, one camera pixel corresponded to an ∼80 nm square in the plane of the gel surface. The bead displacement, Δ*x*, due to *τ* was evaluated by applying a Matlab code to a pair of images taken with 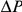 on and off in a region with >100 beads. The code calculated a displacement that provided the best matching between the two images [35]. The error in the displacement was estimated as <0.5 pixels (<40 nm). (We note that this cumulative error is expected to decrease with the number of independent measurements and thus can be reduced by taking repeated measurements of displacements of beads at the gel surface, possibly, in combination with a higher pixelate resolution.) The test was repeated with the microscope focused on fluorescent beads on the cover glass surface and with 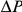 sequentially switched on and off 10 times. No systematic displacement of the beads was detected, indicating that the switching of 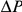 on and off did not shift the cover glass and that the displacement of the beads on the surface of the gel was only due to deformation of the gel. In the measurements of Δ*x* vs. *τ*, we always tried to achieve a bead displacement of at least 10 pixels (800 nm) to have the relative error of Δ*x* at <5%. For *ξ* = 70 µm (typical value), Δ*x* = 800 nm corresponded to a strain 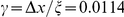 that required 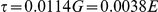. To achieve this value of 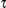 in test channel 2, 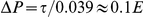
was needed (see the relation between 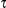 and *E* below). Therefore, the softest gel, 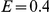 kPa, could be tested with 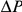 as low as 40 Pa and the application of the highest 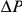
of 30 kPa enabled testing a gel with 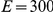 kPa.

Three gels with the highest elastic moduli of those we prepared were sufficiently strong to be subjected to a conventional test, where the extension under tensile stress is evaluated [10]. Slabs of those gels, ∼10 cm long and with cross sections of ∼5×20 mm, were made with a regular pattern of shallow (∼0.8 µm) grooves with a width of 12 µm and a period of 24 µm engraved on their surface (cast from a silicon wafer master mold with an SU8 relief), forming a diffraction grating [36]. The slabs were suspended vertically and loaded by attaching a certain mass, *m*, to their bottom end that resulted in a tensile stress 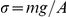, where *A* was the measured cross-section area. The relative extension of central parts of the slabs, 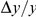, was evaluated by measuring distances between high-order maxima of the diffraction pattern of a laser beam produced by grooves in the middle of the slabs before and after application of the load, 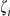 and 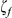, respectively, as 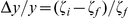. (The measurements of extension in a small area in the middle of a slab minimized the influence of the details of clamping and loading of the slab.) The elastic modulus was calculated from these measurements as 
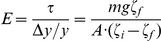
 with an error estimated at <3%. The value of 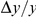 was always <0.10 to minimize the change in *A*, and at this relatively low deformation, the non-linear effects described by a model for a rubber under tension [37], 
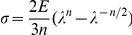
, where 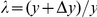 and *n* is a constant on the order 2, were negligible.

## Results

First, we measured the dependence of the maximal flow velocity in the internal regions of the test channels, 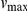, on 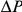. The dependences of 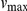 on 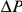 were linear (within ∼3% estimated error of the streakline analysis) for all three test channels for 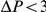 kPa, and we used linear fits to 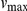 vs. 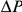 to calculate the ratios 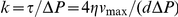 at 0.066, 0.039 and 0.0198 for the test channels 1, 2, and 3, respectively. We also tested variations of the depth of test channel 2 with 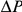 by focusing the microscope objective (20×/0.75) at 100 nm fluorescent beads bonded to the bottom and top of the test channel in the measurement area and calculating differences in readings of the *z*-axis knob at different 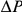. The measured channel depth, 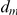, increased by up to 17 µm at 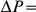
30 kPa, corresponding to a relative change of 10.3%. The cause of the depth increase was the flexibility of the PDMS chip and cover glass and a pressure of ∼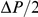 above the atmospheric pressure in the measurement area (the outlet pressure was always nearly equal to the atmospheric pressure). The growth of the channel depth also resulted in faster than linear increase of 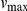 with 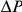. To partially correct for the depth variations, the substrate shear stress at 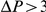
kPa was calculated as 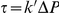, with a pressure dependent coefficient 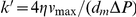 that was increasing nearly linearly with 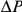, reaching ∼1.13*k* at 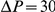 kPa. The cumulative error in *τ* due to combined uncertainties in 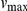, *η* (due to limited temperature control), and the channel depth was estimated as ∼5% at 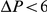 kPa and <10% for 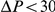 kPa.

**Figure 2:**
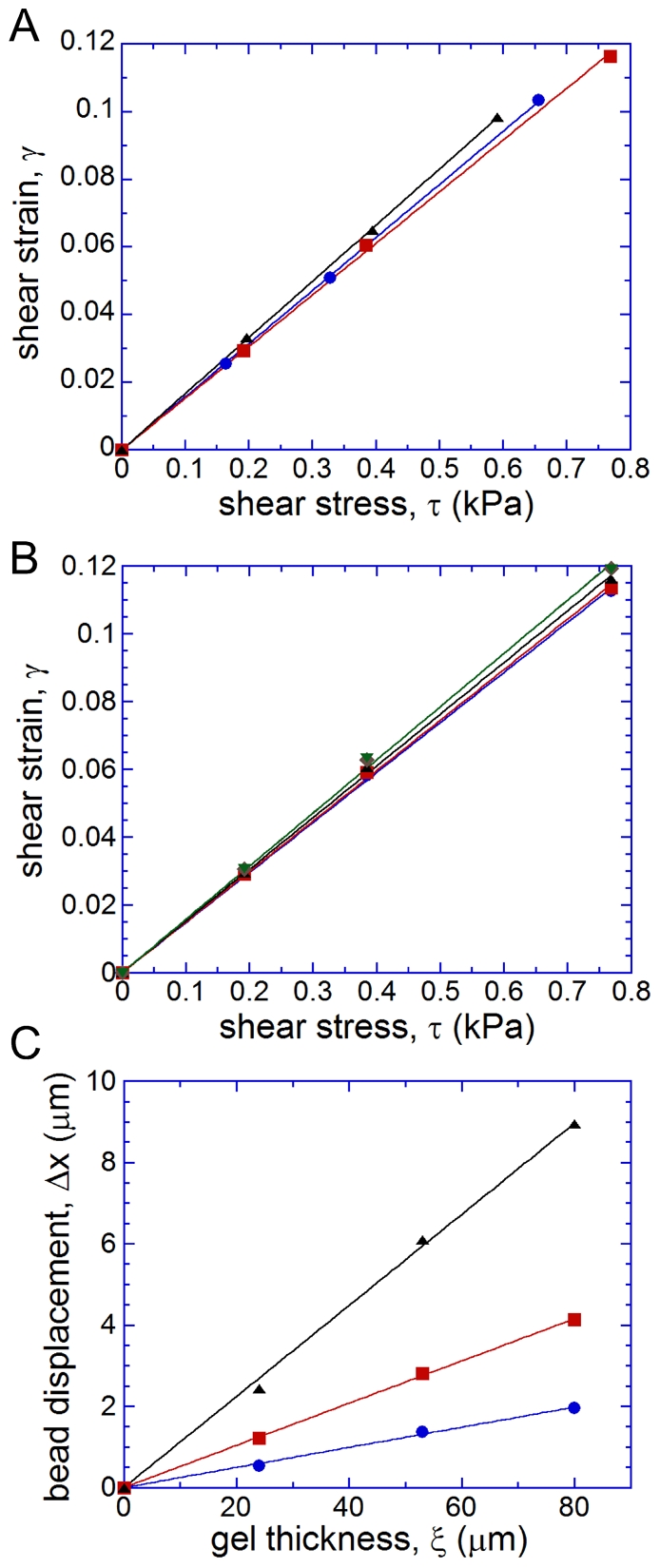
Measurements of gel elastic moduli using the microfluidic device. (a) Gel shear strain, *γ*, as a function of the hydrodynamic shear stress, *τ*, in the internal regions of the test channels 1 (blue circles), 2 (red squares), and 3 (black triangles), for a 70 µm layer of gel with B/C = 45. The data points for the three test channels are fitted with zero-crossing straight lines of matching colors. (b) *γ* as a function of *τ* measured in test channel 2 in five microfluidic devices bonded to gel layers on five separate cover glasses. Gel thicknesses were 58, 58, 64, 65, and 66 µm, all prepared using the same pre-polymer with B/C = 45. Data points for different gel layers are shown by different colors and symbols (circles, squares, diamonds, and triangles) and fitted by separate zero-crossing straight lines. (c) Displacement of beads on the gel surface, 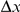, as a function of the gel thickness, *ξ*, measured with microfluidic chips bonded to three different gel layers in test channel 2 at pressures, 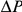, of 1.5 kPa (blue circles), 3 kPa (red squares), and 6 kPa (black triangles), corresponding to 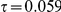, 0.117, and 0.243 kPa, respectively. The three dependencies are fitted with zero-crossing straight lines of matching colors. All three gel layers were prepared from the same pre-polymer with B/C = 58.

To evaluate the consistency and reliability of the technique, we performed a series of measurements on silicone gel layers with 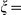
60–70 µm prepared by mixing the B and C components of Sylgard 184 at various proportions. Measurements of 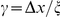 vs. 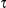 (Fig. 1B) in the three test channels of the device mounted on a gel with 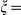
70 µm and B/C = 45 were in good agreement with each other (Fig. 2A). Importantly, tracer beads on the gel surface under the PDMS chip and near corners of the test channels, where 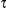 approached zero, did not have any measureable displacement even at 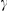 as high as 0.1 (corresponding to 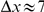 µm) in the central area of a test channel, indicating that the measured bead displacement, 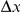, was caused by the local shear stress rather than other factors (such as displacement of the PDMS chip as a whole). The shear modulus of the gel, *G*, as found from zero-crossing linear fitting of 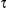 vs. 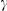 in the test channels 1, 2, and 3 was at 6.37, 6.58, and 6.02 kPa, respectively, corresponding to an average *E* of 19 kPa with a coefficient of variation of ∼5% between the three test channels. Overall, we found test channel 2 to be optimal for the measurements of *E*, because of sufficiently large value and good uniformity of the gel surface displacement, 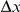, and uniformity of the mid-plane flow velocity.

To further check the reliability of the proposed technique and to test variability of *E* for gel layers from a single batch, we repeated the measurements in test channel 2 in 5 microfluidic devices bonded to gel layers on 5 separate cover glasses. All cover glasses were spin-coated at a speed of 1500 rpm with a gel pre-polymer mixed at B/C = 45 (same ratio as before) and all gel layers were cured simultaneously at identical conditions (2 hr in a 100°C oven). The gel thickness was 62.0±3.5 µm (mean±SD). The dependencies of 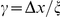 on *τ* were similar for all gels (Fig. 2B), with a mean reciprocal slope, 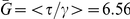 kPa, and a standard deviation 0.18 kPa (3% coefficient of variation), corresponding to *E* = 19.7±0.6 kPa. The high consistency of the values of *E* obtained with identically machined but physically different microfluidic devices in a series of independent tests signified general robustness and reliability of the proposed technique. The results also indicated that gel layers prepared with the same protocol from the same pre-polymer have practically identical elastic modulus.

We also prepared and tested silicone gel layers with B/C = 58 and with 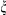 of 24, 53, and 80 µm that were obtained by spin-coating cover glasses at 4000, 2000, and 1250 rpm, respectively (Fig. 2C). The dependencies of 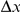 in test channel 2 on 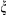 were well fitted by zero-crossing straight lines at all three values of 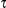 that were tested, 0.059, 0.117, and 0.243 kPa, suggesting thickness-independent constant strain at constant stress. The slopes of the lines corresponded to 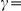
0.025, 0.052, and 0.112, respectively, and 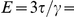
7.1, 6.8, and 6.5 kPa (∼5% coefficient of variation in *E*). The results of this test indicate that the gel layers on the cover glasses have homogeneous mechanical properties that are independent of their thickness. Overall, the results in Fig. 2B and 2C suggest that a large lot of gel-coated cover glasses can be prepared with gel thicknesses appropriate for live cell experiments (usually ∼30 µm) and with practically uniform *E* and that the value of *E* can be measured with the proposed technique using a single cover glass from the same lot. This last cover glass can then have a gel thickness optimized for the measurements of *E* (∼70 µm for large 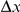 and reduced error in *γ*).

**Figure 3:**
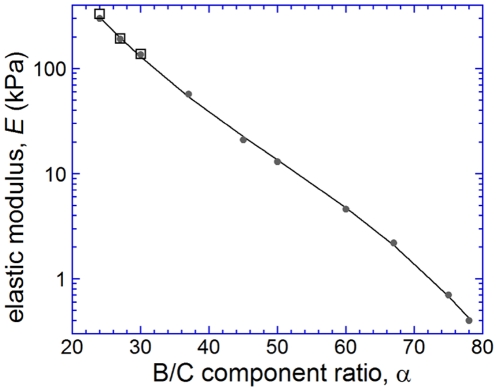
Elastic moduli of gels prepared from Sylgard 184. Elastic modulus, *E*, of silicone gels prepared by mixing the base (B) and cross-linker (C) components of Sylgard 184 as a function of the component mass ratio, *α* = B/C. Grey circles are data points from measurements on thin gel layers with the microfluidic device; black squares are data points from tensile tests on gel slabs; continuous line is a fit of the data points to equation 1, 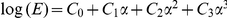, with *E* measured in kPa and with *C*_0_, *C*_1_, *C*_2_, and *C*_3_ equal to 4.86, −0.135, 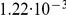, and 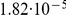, respectively.

We used the proposed technique to measure the elastic moduli of gels prepared from the B and C components of Sylgard 184 mixed at different ratios, *α* = B/C (Fig. 3). After appropriate curing (2 hours in a 100°C oven), the mixtures with *α*≤78 formed solid materials, which responded to small shear stresses by linear deformations and recovered their original shapes after shear stress was removed (flow switched off). (Mixtures with *α*>78 did not solidify properly.) The lowest value of *E* we were able to achieve was 0.4 kPa at *α* = 78. The measurements were feasible for *E* up to ∼300 kPa, corresponding to *α* = 24. (For lower *α* and higher *E*, the substrate deformation 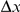 achieved at highest 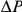
of 30 kPa was too small to be reliably measured, whereas higher 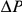 caused excessive deformation of the PDMS chip and compromised the integrity of the device.) Overall, *E* rapidly decayed with *α* following a nearly straight line in semi-logarithmic coordinates. The dependence of 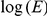 on *α* was well-fitted by a third-order polynomial:
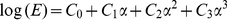
(1)with *E* measured in kPa and with the coefficients *C*_0_, *C*_1_, *C*_2_, and *C*_3_ equal to 4.86, −0.135, 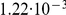, and 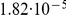, respectively. We note that the measured values of *E* were ∼2 times lower than the previously reported values for Sylgard 184 with B/C ratios between 35 and 55 [15] that could be due to batch-to-batch variability or different curing conditions.

To further validate the proposed technique, we applied known tensile stresses, *σ*, to slabs of the three most rigid gels, with *E* estimated from the shear flow tests at 135, 190 and 300 kPa (Fig. 3), measured the resulting strain, 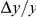, and calculated their elastic moduli as 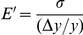. The results agreed well with the shear flow tests. The relative difference between the elastic modulus values obtained with the two methods, 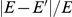, had an average value of only ∼5% for the three gels, well within the estimated errors of the two techniques. (Whereas a similar tensile stress test has been successfully applied to PAA gels with *E* as small as ∼5 kPa [10], we found it difficult to measure the elastic moduli of silicone gels with *E*<100 kPa under tension.)

We preliminary tested aging of the silicone gels by reexamining elastic moduli of 6 randomly selected gel layers on cover glasses that were stored for different time durations (Table 1). We found practically no changes (<12% at ∼7% measurement precision) in gels with B/C = 30, 45, and 60 and *E* = 84, 20, and 3.6 kPa, respectively, after up to 8 months of storage (that was consistent with a previous report [15]). The elastic moduli of the two softest gels, with initial *E* = 1.5 and 0.8 kPa, significantly increased (by ∼110%) after they were stored for 6 months under ethylene glycol. It may be possible to reduce these changes in *E* of the softest gels by optimization of their conditions of preparation (time and temperature of curing) and storage (freezing, no liquid, or immersion into an appropriate storage medium).

**Table 1:** Changes in the elastic moduli of different silicone gels over time.

B/C ratio	Storage time	*E*, initial	*E*, final	% change
30	8 months	84	87	3%
45	3 months	22	21	−5%
45	8 months	19.6	18	−9%
60	8 months	3.6	4.1	12%
67	6 months	1.5	3.2	113%
75	6 months	0.8	1.7	112%

Elastic moduli, *E*, of 6 randomly selected silicone gels prepared by mixing the B and C components of Sylgard 184 were reexamined with the proposed microfluidic technique after various storage times, and the percentage change in *E* was calculated.

Chemical compatibility of silicone gel substrates with mammalian cells has been demonstrated by several groups [13], [38]. Commercial silicones, including Sylgard 184, may contain some low-molecular components that can be harmful for cells. Nevertheless, in our pilot tests with human umbilical venous endothelial cells (HUVECs; by Lonza, Basel, Switzerland) and mouse embryonic fibroblasts (MEF; isolated and cultured as previously described [39]) plated on silicone gel layers made of the Sylgard 184 components and coated with fibronectin, we did not observe any cell abnormalities, when gel-coated cover glasses were incubated in a buffer solution for ∼1 hour prior to cell plating (see also [40]). (The relatively small thickness of the gel layers was a likely factor facilitating rapid elution of potentially harmful compounds from the gels.)

## Discussion

The proposed technique for measurements of elastic moduli of thin gel layers has several advantages over the use of AFM. First, the proposed technique does not require any costly special instruments. The measurements of the gel deformations are performed with a basic fluorescence microscope that would normally be available in a laboratory studying cell rigidity sensing and performing TFM. The only special treatment of the gel is the attachment of fluorescent beads to its surface. However, these 40 nm far-red fluorescent beads are of the exact same type as often used in TFM studies [41], [42]. (The far red spectrum of the beads minimizes their overlap with many fluorescent tags used in cell biology.) Moreover, the treatment of the gels with 3-aminopropyl trimethoxysilane and 1-Ethyl-3-(3-dimethylaminopropyl) carbodiimide (EDC) that we utilized to covalently link beads to their surface also facilitates coating the surface with extracellular matrix (ECM) proteins that mediate the adhesion of animal cells. Specifically we used this treatment to coat the gel surface with fibronectin for plating of HUVEC [40] and MEF. The 40 nm beads on the interface between the cover glass and the gel, which are not necessary for TFM, can be omitted from the gel preparation protocol at expense of a greater error in the measurement of the gel thickness, or a dedicated cover glass for measuring *E* can be prepared from the same silicone gel batch.

Second, the analysis of results of AFM measurements is complicated by multiple factors. For a finite gel thickness, *ξ*, the expected dependence of the depth of indentation on the force applied to the probe is a complex non-linear function of *E*, *ξ*, and the radius of curvature of the tip of the probe [19]. Standard commercial AFM probes have sharp tips that are poorly suited for gels, because the measurements are very sensitive to the exact shapes of the tips. In addition, the application of even a relatively small force may result in excessive deformations of the gel substrate, beyond the linear elasticity regime, especially when *E* is low. Customized AFM probes, with a microsphere glued to the tip, produce substantially better results [19]. Nevertheless, stiffness of the AFM probe and the diameter of the microsphere need to be carefully calibrated, and the possibility of imperfect loading conditions and the difficulty in identifying the point of the first contact between the probe and the gel remain significant sources of possible experimental errors [19], [43]. Therefore, the errors in the measurements of *E* with AFM are often not stated and validations by extensional tests of gel slabs are frequently performed.

Unlike AFM measurements, the proposed technique relies on linear equations directly following from the first principles of continuum mechanics, 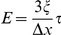, and low Reynolds number fluid mechanics, 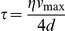, with *η* being a known viscosity of the working liquid and with all other parameters measured under a fluorescence microscope either directly (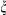, 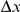, and *d*) or from the analysis of steaklines (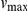). In this respect, the proposed technique resembles the measurements on bulk gel samples under extension or shear [10], [20]. In addition, a displacement of the top of the gel 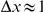 µm, corresponding to a shear strain 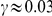 for a 30 µm thick gel, is sufficient to measure 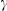 with ∼5% accuracy. Therefore, the measurements can always be performed safely within a linear elasticity regime and with 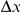 close to those generated by cells. Importantly, the experimental uncertainties (errors) of values of all of the parameters can be readily estimated. The largest errors are in the evaluation of *η* (∼3% because of temperature variations), measurements of 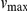 (up to 3%), and in the measurements of 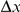 (up to 5%), resulting in an estimated 5–10% error in *E*. This error estimate is consistent with the discrepancies between the values of *E* measured in the microfluidic device and in extensional tests for the three most rigid gels (Fig. 3).

Third, the proposed technique made it possible to measure the elastic moduli of gels in a range of nearly 3 orders of magnitude, from 0.4 to 300 kPa, in a small-strain linear regime using a single microfluidic device and flow-control setup. The broad range of *E* with the proposed technique is enabled by the broad range of 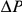. At 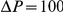 Pa that can be reliably applied (1% error at 1 Pa resolution), the stress in test channel 2 is 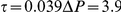 Pa, resulting in 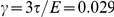 (which is expected to be well in linear regime) in the gel with the lowest *E* of 0.4 kPa, whereas the maximal 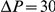 kPa is sufficient to measurably deform a gel with *E* = 300 kPa. Moreover, the AFM measurements become increasingly difficult as *E* is reduced to 1 kPa and less, because of small force applied to the probe, the danger of excessive deformation of the gel, and a combination of interactions between the gel and the probe unrelated to gel rigidity [27]. In contrast, the proposed technique has the smallest error when applied to gels with lowest *E*. Indeed, low values of *E* (<1 kPa) make it possible to operate the device at 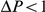 kPa, resulting in negligible deformation of the test channels, while eliciting large 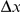, thus minimizing the errors in the evaluation of 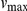 vs. 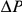, 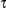 vs. 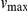, and 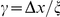. The control of flow in the microfluidic test channels enables applying a small and nearly uniform shear stress to a large area of a gel that is an optimal way of testing the mechanical properties of gels with low *E*.

The proposed microfluidic technique may also be applicable to measurements of *E* of thin layers of PAA gels, if an appropriate method is found to attach microfluidic chips to PAA gel-coated cover glasses without causing excessive deformation of the gels. Plausible options here are the application of magnetic forces pushing the chip against the cover glass (magnetic clamping) [44] and surrounding the test channels with a large-area groove and applying regulated vacuum to it (vacuum clamping) [45].

Silicone gels present an attractive alternative to PAA gels and other hydrogels as materials to study cell rigidity sensing and perform TFM of adherent cells primarily because they are not susceptible to drying and do not have much sensitivity to the ionic content or pH of aqueous media. In addition, our preliminary test showed practically no variation of the elastic modulus (aging) of silicone gels with *E*≥3.6 kPa after as much as 8 months storage, whereas the aging of hydrogels over the same time period can be substantial [43]. The aging of silicone gels might be further reduced by hard-baking them at 150–200°C [18], [46]. Sylgard 184 has very low autofluorescence [47], and the covalent binding of tracer particles to the silicone gel surface applied here (see also [16]) makes it possible to measure the displacement of the gel surface under wide-field (non-confocal) fluorescence illumination with high resolution and minimal background. As compared to the imbedding of tracer beads into the bulk of a gel, as often practiced with PAA gels [42], the placement of beads on the gel surface has an additional advantage of eliminating the uncertainty in their vertical (*z*-axis) position and thus improving the accuracy of the conversion of maps of bead displacement into maps of cell traction forces in TFM [40]. TFM on silicone gel substrates has been demonstrated by several groups [13], [38], [40], and the gel surface treatment for binding the beads can be readily used to coat the surface with various ECM molecules suitable for animal cells [40].

Among other advantages of silicone gels is the demonstrated possibility to micro-patterning their surfaces [13], [15] and their high refractive index. It is ∼1.41 for Sylgard 184 used in this study and reaches ∼1.49 for other silicone gels [40], making it possible to combine the TFM with total internal reflection fluorescence (TIRF) microscopy to visualize the areas where cells adhere to the substrate and to correlate the maps of traction forces and cell adhesion areas [16], [40], [48]. Our work also shows that cover glasses coated with silicone gel layers can be easily used as substrates for microfluidic chips, opening a way to applying a variety of microfluidic techniques to cells on soft gel substrates.

This study demonstrates that silicone gel layers with thicknesses suitable for high-resolution microscopy and with elastic moduli covering nearly the entire physiological range (0.4–300 kPa) can be easily prepared from an inexpensive and widely used Sylgard 184 kit. Even with inevitable variability between different batches of Sylgard 184, the master curve of the dependence of *E* on the ratio of the two Sylgard 184 components (Fig. 3) should provide good initial guidance for future cell biology applications. Moreover, we showed that gels prepared from the same Sylgard 184 pre-polymer have nearly uniform values of *E* (Fig. 2B). Therefore, if an uncompromised accuracy in *E* is desired, a suitable experimental protocol could be to prepare a large lot of cover glasses with gel layers from the same Sylgard 184 pre-polymer, measure *E* of one or several gel samples using the proposed technique, and consistently use the rest of the lot through an entire series of experiments on cells.

To summarize, we have developed a new technique for measurements of elastic moduli of thin gel layers on cover glasses based on the application of known hydrodynamic shear stresses in a microfluidic device. The proposed technique does not require any expensive specialized equipment, can be applied to gels with a broad range of elastic moduli, and has small and simple to estimate measurement errors. We applied this technique to measure the elastic moduli, *E*, of gels obtained by mixing two components of a widely used commercial silicone, Sylgard 184, establishing a master curve of *E* for different mixtures, and showing that these mixtures cover the entire physiological range of *E*. We also showed that a large lot of gels with consistent elastic modulus and thickness can be prepared. We believe that this work will lead to broader use of silicone gels in cell traction force microscopy and rigidity sensing studies.
